# Thermodynamics of Adsorbed Methane Storage Systems Based on Peat-Derived Activated Carbons

**DOI:** 10.3390/nano10071379

**Published:** 2020-07-15

**Authors:** Ilya Men’shchikov, Andrey Shkolin, Elena Khozina, Anatoly Fomkin

**Affiliations:** Dubinin Laboratory of Sorption Processes, Frumkin Institute of Physical Chemistry and Electrochemistry, Russian Academy of Sciences, Leninskii Prospect 31, Building 4, 119071 Moscow, Russia; shkolin@bk.ru (A.S.); khozinaelena@gmail.com (E.K.); fomkinaa@mail.ru (A.F.)

**Keywords:** activated carbon, high-pressure methane adsorption, thermodynamics of adsorption systems

## Abstract

Two activated carbons (ACs) were prepared from peat using thermochemical K_2_SO_4_ activation at 1053–1133 K for 1 h, and steam activation at 1173 K for 30 (AC-4) and 45 (AC-6) min. The steam activation duration affected the microporous structure and chemical composition of ACs, which are crucial for their adsorption performance in the methane storage technique. AC-6 displays a higher micropore volume (0.60 cm^3^/g), specific BET surface (1334 m^2^/g), and a lower fraction of mesopores calculated from the benzene vapor adsorption/desorption isotherms at 293 K. Scanning electron microscopy (SEM), X-ray diffraction (XRD), and small-angle X-ray scattering (SAXS) investigations of ACs revealed their heterogeneous morphology and chemical composition determined by the precursor and activation conditions. A thermodynamic analysis of methane adsorption at pressures up to 25 MPa and temperatures from 178 to 360 K extended to impacts of the nonideality of a gaseous phase and non-inertness of an adsorbent made it possible to evaluate the heat effects and thermodynamic state functions in the methane-AC adsorption systems. At 270 K and methane adsorption value of ~8 mmol/g, the isosteric heat capacity of the methane-AC-4 system exceeded by ~45% that evaluated for the methane-AC-6 system. The higher micropore volume and structural heterogeneity of the more activated AC-6 compared to AC-4 determine its superior methane adsorption performance.

## 1. Introduction

Storage of adsorbed natural gas (ANG) represents an effective alternative to the known techniques of liquefied and compressed gas due to several reasons, such as high specific capacity, safety, and energy efficiency [1,2,3]. In most studies, when choosing an adsorbent for an ANG system, the most crucial criterion is the methane adsorption capacity [2,3,4]. Among adsorbents, activated carbons (AC) [2,4,5,6,7,8], metal-organic frameworks (MOF) [5,9,10], and porous organic polymers (POP) [10,11] are the most promising for use in ANG technology because of their high adsorption capacity of methane, which is the major component of natural gas. It should be noted that the utilization of these materials for the ANG storage systems implies that they meet the requirements imposed on these systems. Among these requirements are chemical stability, mechanical strength, thermal stability, conductivity, minimized affinity to water, and heavier natural gas components (ethane, propane, and butane), accessibility and simplicity of the synthesis, and low costs of reactants and final product [2,9]. Although MOFs show high promise in this area, their use in the loose powder form with low packing density produced by most conventional synthetic techniques is not practical in the ANG systems, and, therefore, they must be shaped [12,13]. Shaping or densification of MOFs may cause degradation of their porous structure due to their low resistance against mechanical loads [14], which leads to a deterioration in the methane adsorption capacity [15]. Moreover, Zhang et al. [16] found that the most promising HKUST-1 adsorbent lost 32% of its adsorption capacity over 200 sorption/desorption cycles due to the irreversible blocking of almost half of the pore volume. However, despite the advances in developing new synthetic technologies, the large-scale production of MOF- and POP-based adsorbents is limited by a low level of reproducibility between different batches of final product and high costs of the production process [10,12,17].

Therefore, for the foreseeable future, microporous ACs with their unique properties, such as high thermal stability and mechanical strength [2,5,8], remain most suitable for the ANG storage technique. Additional properties of ACs, which are essential for the ANG systems, are the high adsorption capacity per unit volume, preferential adsorption of gas in the presence of moisture (hydrophobicity), low resistance to gas flow, and complete release of adsorbates at increasing temperatures and decreasing pressures [18]. As shown in the experimental study of methane adsorption in a series of commercial (Maxsorb, Filtrasorb 400, NuCharRGC300) and laboratory synthesized ACs with various pore size distribution and MOF (HKUST-1), a properly designed carbon adsorbent with an optimal micropore/mesopore ratio demonstrates an excellent adsorption performance at high pressures without any loss in the porosity [5].

When searching for an efficient carbon adsorbent for methane storage, an important criterion is the availability of raw materials. Peat, matching this criterion, is a partially carbonized phytomass (up to 61%), and it is considered a suitable raw material for the production of carbonaceous adsorbents in terms of energy consumption and lows cost [19,20]. As a fossil raw material with an extensive capacity, peat finds use as a precursor for the synthesis of promising adsorbents for many applications, including ANG technology. From this standpoint, it is crucial to determine the influence of both raw material and activation conditions on the porous structure, chemical composition, and morphology of the final product—activated carbon—and its adsorption properties relative to methane. The adsorption performance of a porous material with respect to a gas can be predicted based on the structural and energy characteristics (SEC) calculated from data on standard vapor adsorption according to the Dubinin theory of volume filling of micropores (TVFM) [21]. Therefore, it is essential to establish a relationship between the methane adsorption capacity of peat-derived adsorbents and their SEC values, which, in turn, are affected by chemical composition and morphology inherited from peat and methods of their production [22].

It should also be noted that a proper design of ANG storage facilities operating within a wide range of temperatures and at high pressures requires accounting for both the heat release/absorption and deformation of an adsorbent upon the adsorption/desorption processes. These phenomena have a significant impact on the thermodynamic characteristics of gas adsorption systems at high pressures [23]. Many authors calculated the heat of adsorption by substituting various models of adsorption [24,25] into the well-known Clausius–Clapeyron equation, in which an adsorbate in its gaseous phase is endowed with ideal gas-phase behaviors.

An approach for calculating the thermodynamic functions of adsorption systems, which takes into account the effects associated with the nonideality of an equilibrium gas phase and non-inertness of an adsorbent at high pressures, has been developed by Bakaev [26,27]. Behind this approach is the concept by Guggenheim that the thermodynamics of the adsorption equilibrium is rigorous if the thermodynamic functions of an adsorption system are evaluated using only the quantities, which are measured experimentally [28,29,30]. Using the methods of replacement of variables and Jacobian-determinant in thermodynamics of adsorption equilibrium, Bakaev derived an equation, which is recognized as a complete formula for calculating the differential molar isosteric heat of adsorption [26]. This approach makes it possible to neglect the surface effects and avoid the difficulty in interpreting adsorption data associated with the Gibbsian treatment of adsorption phenomena in terms of interfacial excess amounts [31].

The applicability of the Bakaev equation was demonstrated by calculating the thermodynamic functions characterizing the adsorption of gases (Xe, Kr, Ar, N_2_, O_2_, H_2_, CH_4_, CO_2_, and He) in microporous adsorbents (zeolites and ACs) in a wide range of temperature and pressure [32,33,34]. It should also be noted that an analysis of thermodynamic parameters of an adsorption system, as a function of pressure, temperature, and amounts of adsorbed methane, also provides information on changes in the state of methane molecules in micropores [32]. Indeed, in [5], an advantage in the performance of ACs in terms of the working capacity compared to HKUST-1 was attributed to a lower isosteric heat of adsorption at the early adsorption stages.

The objective of the present work is to evaluate and compare the SEC values of two ACs, which were produced from the same raw material as peat, but at different activation durations, from the standard adsorption measurements by TVFM equations. The second task is to find a correlation between the SEC values of the AC samples and data on their chemical composition and morphology obtained by independent structural methods as X-ray diffraction (XRD), small-angle X-ray scattering (SAXS), and scanning electron microscopy. Our study also focuses on the thermodynamic functions derived qualitatively for methane adsorption in two peat-derived ACs measured at pressures up to 25 MPa and over the temperatures from 178 to 360 K, i.e., both in sub- and supercritical states. The data are essential for identifying the optimal thermal conditions of such a system, including the charge/delivery processes.

## 2. Materials and Methods

### 2.1. Adsorbent

Shredded high-grade metamorphic peat from Central Russia (supplied by JSС Electrostal Research and Production Association “Neorganika”, Electrostal, Russia) was used as a precursor for the production of the activated carbons in three stages, including carbonization, thermochemical activation, and steam activation.

After passing the preliminary treatment of crushing and separation, peat was impregnated with potassium sulfide (K_2_SO_4_, > 99.0%, Sigma-Aldrich, St. Louis, MO, USA) and mixed to form a homogeneous elastic paste. Afterward, the paste preform was pressed and granulated with the use of extruding jets. The cylindrical granules thus obtained were dried and carbonized in a drum furnace at the temperatures of 873−973 K in order to remove both the residual water and volatile compounds, and decompose the peat organic matter.

Then the granulated material was activated in a rotary furnace at the temperature within a range of 1053−1133 K for 1 h. At this stage, the volatile compounds were completely removed. As a result, the material is enriched with carbon, which provides its mechanical strength and density. The activation procedure gives rise to a porous structure in the carbonizate [20]. Due to the peculiarities of the production process of carbon adsorbents from peat with the complex chemical composition, the activated granules can contain up to 30 wt.% potassium, 10% sulfur, and up to 10 wt.% ash components [35].

For this reason, the activated granules were cooled, and then they passed through a special washing series including leaching with returned alkali solutions, washing in water and hot hydrochloric acid (EKOS 1, Staraya Kupavna, Moscow Region, Russia), and a final rinsing with water. In the end, the wet granules were placed into a tumble drier to reduce moisture content up to 3−5 wt.%.

As follows from [19,20,36], the optimal temperature of heat treatment of carbon-containing biomass ranges from 1073 to 1173 K. In order to develop microporosity, the granules were subjected to additional steam activation carried out in a reactor at 1173 K. It is known that at a particular temperature of activation, weight loss due to burn-off (or degree of activation) increases with activation duration producing micropores up to excessive activation, resulting in an increase in transport pores and macropores [36,37] and a decrease in the characteristic energy of adsorption [21]. According to [38,39], the burn-off of 30−40% matches the development of a maximum microporosity in activated carbons. We attained these values at the activation times varied from 30 to 45 min. By using two different activation duration, 30 and 45 min, we obtained two peat-based AC samples labeled as AC-4 and AC-6, respectively. Due to the shorter duration of this stage, AC-4 had a lower degree of activation compared to AC-6.

### 2.2. Adsorptive

The adsorptive gas used in the experiments was high purity (99.999%) methane. Methane has the following physicochemical properties: molecular mass *M* = 16.0426 g/mol; boiling temperature *T*_0_ = 111.66 K; critical temperature *T*_cr_ = 190.77 K; critical pressure *P*_cr_ = 4.641 MPa [40].

### 2.3. Methods

Porous characteristics of the activated carbons were estimated from the isotherms of benzene standard vapor adsorption at 293 K measured by employing an original gravimetric vacuum adsorption setup designed in IPCE RAS [41,42]. A maximal measurement error was ± 0.19 mmol/g with a confidence level of 99% determined according to [43]. A LOIP LT-411 thermostatic bath circulator and a LOIP FT-311-80 cryostat (ultralow temperatures) were used for temperature control with an error of ± 0.01 K. The SEC values of the carbons, such as a specific volume of micropores *W*_0_, standard characteristic energy of adsorption *E*_0_, and effective half-width of micropores *x*_0_, were evaluated by applying the Dubinin–Radushkevich (D-R) equation [21]. The Brunauer–Emmett–Teller (BET) method [44] was used to calculate specific BET surface *S*_BET_ from the data on benzene adsorption at 293 K with a benzene molecular area of 0.40 nm^2^ for a flat molecular orientation in a monolayer [45]. The specific surface area of mesopores (*S*_meso_) was calculated from the benzene adsorption/desorption data using the well-known Kiselev equation [46]. The specific mesopore volume was calculated as *W*_meso_ = *W*_S_ − *W*_0_, where *W*_S_ is the total pore volume calculated from the amount of benzene adsorption at the relative pressure *P*/*P*_s_ = 0.99.

The surface morphology and elemental composition of mechanically ground AC-4 and AC-6 were examined by scanning electron microscopy (SEM) using a Quanta 650 FEG microscope (FEI Company, Hillsboro, OR, USA) equipped with an Oxford Energy Dispersive X-ray (EDX) detector operating at 30 kV accelerating voltage. The data on the elemental composition of both samples were obtained by averaging ten measurements.

The phase composition of AC-4 and AC-6 was examined by powder X-ray diffraction (XRD) with an Empyrean Panalytical (Panalytical BV) diffractometer in Bragg–Brentano geometry using Ni-filtered CuKα-radiation (λ_Cu_ = 0.1542 nm) in the 2θ angular range of 10–120 degrees. The samples were ground to powder, and no binder was employed. The ICDD PDF2 database was used for phase identification. The detailed qualitative analysis was carried out through the graphite characteristic reflections—(002), (10), (100), (101), and (11).

The results from benzene adsorption analysis based on TVFM were compared with SAXS data on the porous structure of ACs obtained at a SAXSess diffractometer (Anton Paar GmbH, Graz, Austria). Powdered samples were measured in transmission geometry in a vacuum chamber. Monochromatic Cu-Kα radiation and a 2D Imaging Plate detector were employed, and the range of scattering q vectors was from 0.1 to 27 nm^−1^.

Methane adsorption equilibria on the AC-4 and AC-6 adsorbents were studied within a range of pressures from 5 Pa to 25 MPa and at the temperatures varied from 178 to 360 K by the volumetric-gravimetric method using three original adsorption devices developed in IPCE RAS and described in detail in our previous works [47,48,49]:

- a semi-automatic adsorption weight vacuum unit (from 5 Pa to 0.1 MPa, gravimetric method; accuracy of ± 1.5%) [47];
- a universal adsorption-dilatometer setup (0.1−6 MPa, volumetric method, accuracy of ± 3%) [48];
- an original volumetric-gravimetric high-pressure set-up (0.2−25 MPa, accuracy ± 5%) [49].

The values of methane adsorption *a* were determined as the total content of adsorbed methane in micropores of an adsorbent:

(1) $$a=\left(V-V_{a}\right)\rho_{g}/m_{0}$$

where *V* is the total geometric volume of the system, *V*_a_ is the volume of an adsorbent with micropores, ρ_g_ is the density of gaseous phase at given pressure *P* and temperature *T*, *m*_0_ is the mass of a regenerated adsorbent. As follows from the data below, the specific surface of mesopores in both adsorbents constituted no more than 10% of the total specific surface, and therefore, one can neglect the adsorption processes in mesopores. When calculating the value of methane adsorption *a*, the volume of adsorbent with micropores was determined as a sum of the volume of this adsorbent determined via helium pycnometry, *V*_He_, and product *m*_0_⋅*W*_0,_ where micropore volume *W*_0_ is evaluated from the data on benzene adsorption at 293 K using the D-R equation [21].

## 3. Results and Discussion

### 3.1. Structure and Morphology Characterization

In order to reveal the influence of the activation time on the porous structure of the peat-derived carbon adsorbents, the SEC values and the other parameters characterizing mesopores for AC-4 and AC-6 summarized in Table 1 should be compared.

As follows from Table 1, the steam activation duration had a significant impact for developing the microporosity. It should be noted that ceteris paribus, the additional time of activation of AC-6 was responsible for the development of a specific BET surface through the enlargement of micropores without the formation of a noticeable amount of mesopores. The AC-4 sample is characterized both by the higher energy of adsorption and the proportion of mesopores in the total pore volume compared to AC-6. One can expect that the differences in the porous structure of these two ACs manifest in variations in the methane adsorption capacities.

**Figure 1:**
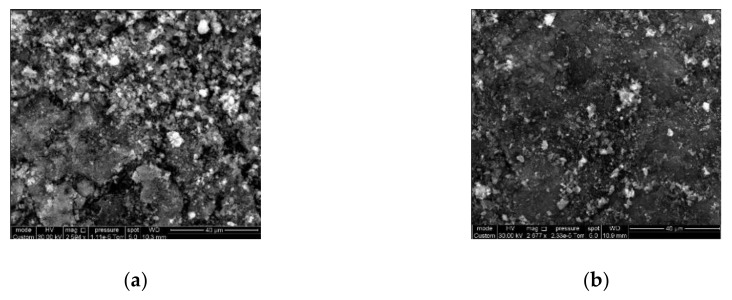
Scanning electron microscopy (SEM) images of the peat-derived AC-4 (**a**) and AC-6 (**b**) adsorbents. The scale bar is 40 µm.

The SEM images (Figure 1a,b) allowed for a qualitative assessment of the surface morphology of AC-4 and AC-6. The images indicate that the carbons are not homogeneous in density. As can be seen from the comparison of Figure 1a,b, the heterogeneous surfaces of the AC-4 and AC-6 powders are characterized by different proportions of relatively dark (dense carbon phase) and light (relatively low-dense carbon phase) areas. The steam activation stage implies a partial burning-off of a non-graphite amorphous phase associated with the light areas in the SEM images. Therefore, the SEM image of more activated AC-6 shows fewer light inclusions compared to AC-4.

The heterogeneous surface morphology of the peat-based adsorbents correlates with the SEM-EDX data on their elemental chemical composition given in Table 2. The diverse chemical composition of the adsorbents is reliant on the precursor (peat), activation process, and activating agent (K_2_SO_4_). Therefore, besides the relatively high percentage of oxygen, especially in AC-6 (see Table 2), the chemical composition of both carbon adsorbents also includes such heteroatoms as Si, Al, Ca, Fe, the presence of which can be attributed to ash and plant residues decomposed under peat-forming geochemical processes [35]. Potassium and sulfur are residuals of the activating agent.

As seen from Table 2, the percentages of carbon in both peat-based adsorbents are less than that found for commercial peat-based Sorbonorit 4 (Norit Ltd., Netherland): 95 wt.% [50], and close to the data for the peat-based ACs prepared via a one-step steam activation process (64−91 wt.%) reported in [51].

When comparing the data on the elemental chemical composition of AC-4 and AC-6 in Table 2, one can conclude that the increase in the steam activation time reduced the relative amounts of carbon, potassium, and sulfur. In AC-6, metal impurities such as Fe and Mg were not found. At the same time, the percentage of unremovable Si and O increased. Thus, the higher activation time of AC-6 caused the higher oxygen/carbon (O/C) ratio: ~0.39 compared to AC-4 (O/C = 0.14).

The presence of heteroatoms (O, K, S, etc.), which form surface functional groups, largely determines the surface chemical characteristics of the carbon adsorbents, thereby affecting their interactions with adsorptive, and as a consequence, the adsorption process, especially in the early stages of adsorption.

**Figure 2:**
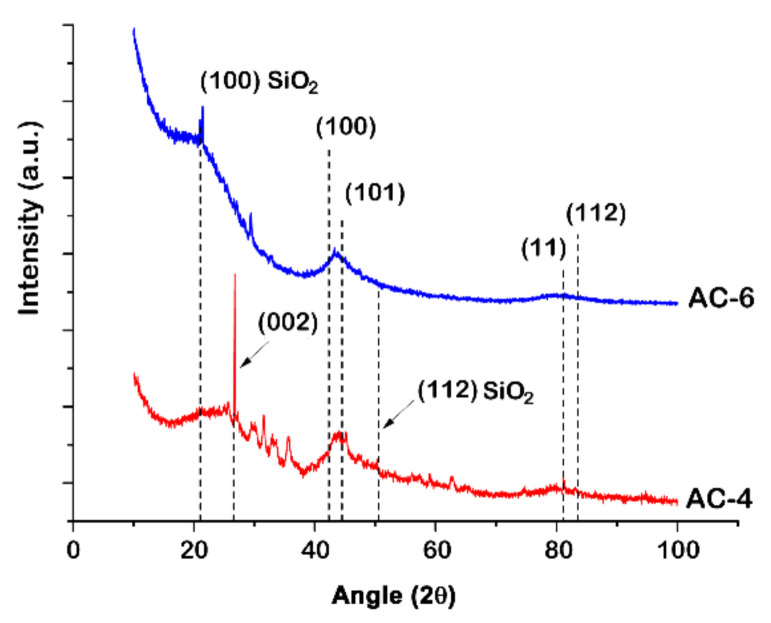
X-ray diffraction (XRD) patterns for the peat-derived activated carbons: AC-4 and AC-6. Dashed lines show the primary reflections of graphite and quartz.

The analysis of powder XRD patterns for both samples (see Figure 2) revealed the pronounced reflections related to carbon species in hexagonal graphite lattice and quartz. The graphite crystallites are likely inherited from peat with a variable degree of thermal metamorphism.

The activation stages promoted the growth and subsequent ordering of the crystalline phases to a certain limit. But over this limit of activation, the peak shapes are somewhat broadened, and their intensity is lowered (compare AC-4 and AC-6), which is indicative of a more amorphous and non-graphitized structure in AC-6. Moreover, the strength of the diffraction intensity of AC-6 at low angles is more significant compared to that of AC-4, which is consistent with a higher density of pores in AC-6 (see Table 1) [52].

**Figure 3:**
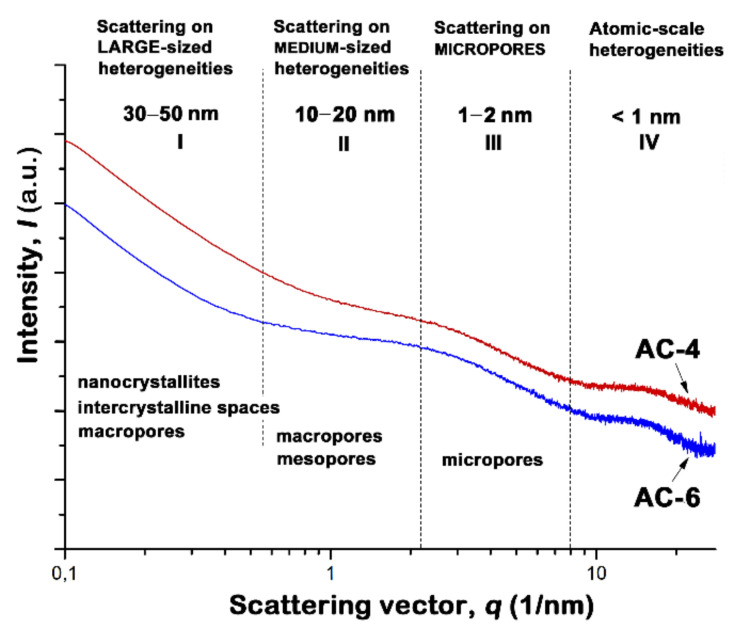
The log-log plot of scattering intensities versus scattering vector q for the peat-derived activated carbons obtained at different steam activation times.

As shown in [53,54], SAXS is able to provide topological information from the molecular to mesopore scale, regardless of the sample crystallinity. Characterization of the carbons by SAXS is shown in Figure 3.

The linear log-log Guinier plots of the scattering intensity *I* on the scattering vector *q* in the region between 2 and 8 nm^−1^ (marked as III in Figure 3) indicate scattering by a monodisperse system [53,54]. Therefore, the values of radius of gyration used for estimations of pore sizes, *R*_G_, can be calculated with the use of the tangent method and Guinier formula for a slit-like model of pores [55]: *q*^2^*I*(*q*) = *I*_0_·exp [−*R*_G_^2^*q*^2^]. We found that the values of *R*_G_ for AC-4 and AC-6 are close enough to each other: 0.48 and 0.50 nm, respectively.

When analyzing the experimental SAXS data for the carbons under study (see Figure 3), we need to compare them with an empirical relationship proposed by Dubinin with coworkers for carbon adsorbents with relatively homogeneous chemical composition and narrow slit-like pore size distribution [56,57,58]:

(2) $$E_{0}R_{G}=14.8\pm 0.6\;kJ\cdot \mathrm{nm}/\mathrm{mol}$$

where *E*_0_ is the characteristic energy of adsorption determined from the adsorption of standard benzene vapors (see Table 1) and *R*_G_ is the radius of gyration of micropores evaluated from the SAXS data.

We found that the values of *E*_0_⋅*R*_G_ determined for AC-4 and AC-6 (10.3 and 9.1 kJ⋅nm/mol, respectively) differ markedly from that in Equation (2). Since the magnitudes of *E*_0_ for AC-4 and AC-6 from the adsorption data are typical for microporous adsorbents [see, for example, Reference 32], we attribute this deviation to an effect of the multi-component chemical composition of peat on the formation of turbostratic carbon nanocrystallites in the final ACs. As a consequence, the variation in the sizes and shapes of carbon nanocrystallites over the sample plus the amorphous phase detected by XRD made it impossible to describe the porous system using a particular model of pore shapes for correct evaluation of gyration radius *R*_G_ from the SAXS data. Thus, the surface chemistry of ACs can be a crucial feature of novel peat-based adsorbents and should be taken into account when developing new adsorption systems. Undoubtedly, the structural data only for two samples are insufficient to establish an unambiguous correlation between the SEC quantities and real structural properties of ACs. Nevertheless, these new findings contribute to establishing a general pattern needed for the synthesis of adsorbents with tailored structure.

### 3.2. Methane Adsorption on the Peat-Derived Carbon Adsorbents

**Figure 4:**
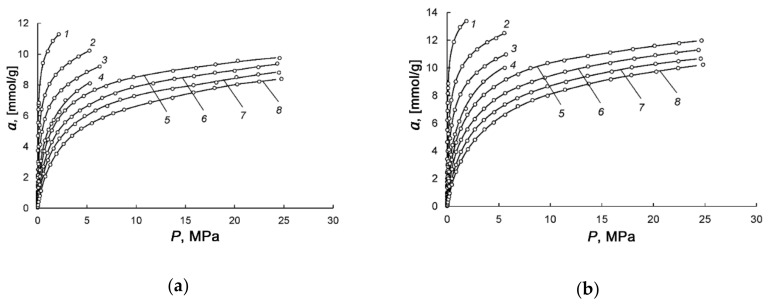
Dependences of methane adsorption in AC-4 (**a**) and AC-6 (**b**) on pressure at temperatures, K: 178 (*1*), 216 (*2*), 243 (*3*), 273.15 (*4*), 300 (*5*),320 (*6*), 340 (*7*); 360 (*8*). Experimental data are shown by symbols, and lines are the approximation of experimental data by Equation (3).

Figure 4 presents experimental isotherms of methane adsorption in AC-4 and AC-6 at the temperatures varied from 178 to 360 K and pressures up to 25 MPa.

We used a formula derived by Bakaev for the total adsorption isotherm [59] to fit the experimental data shown in Figure 4a,b:

(3) $$a\left(P\right)=\frac{k_{0\;}\left(k_{1}P+2k_{2}P^{2}+3k_{3}P^{3}\right)}{1+k_{1}P+k_{2}P^{2}+k_{3}P^{3}}$$

where *k*_0_ characterizes an adsorption system, *k*_1_, *k*_2_, *k*_3_ are the temperature-dependent and numerically adjusted coefficients, and *P* is the equilibrium pressure expressed in Pa.

Equation (3) was successfully used for describing the adsorption process in various adsorbents, including zeolites [59], polymer sorbents [60], and ACs [61]. In our case, the maximum regression error did not exceed 3%, which made it possible to calculate a set of adsorption and thermodynamic parameters of the systems with high accuracy.

It can be seen from Figure 4a,b that over the entire range of temperatures and pressures, the more activated AC-6 with a large volume fraction of micropores shows a higher adsorption capacity for methane compared to AC-4.

**Figure 5:**
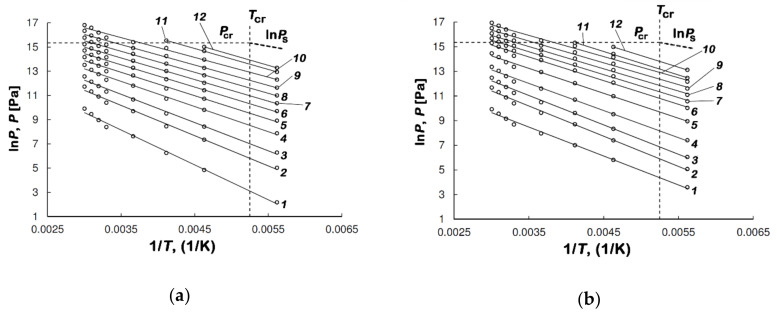
The isosters of methane adsorption in AC-4 (**a**) and AC-6 (**b**) at the values of methane adsorption, mmol/g: 0.1 (*1*); 0.5 (*2*); 1.0 (*3*); 2.0 (*4*); 3.0 (*5*); 4.0 (*6*); 5.0 (*7*); 6.0 (*8*); 7.0 (*9*); 8.0 (*10*); 9.0 (*11*); 9.5 (*12*). Symbols mark the experimental data, and the solid straight lines show the linear function approximation. The bold dashed line shows ln*P*_s_, where *P*_s_ is the saturated vapor pressure; the dashed lines show the critical pressure and temperature of methane.

Figure 5a,b demonstrate the isosteres of methane adsorption in AC-4 and AC-6, which were calculated from the isotherm of methane adsorption shown in Figure 4a,b.

As follows from Figure 5a,b, the isosteres of methane adsorption in AC-4 and AC-6 plotted in ln*P* = *f*(1/*T*) coordinates are well approximated by linear functions. No deviation from the straight lines was observed when passing through the critical temperature of *T*_cr_ = 190.77 K to the nonideality of a gaseous phase. The linearity of adsorption isosteres in the region, where gases show a significant deviation from ideality, is indicative of a specific state of a highly dispersed substance in micropores. This state enables the accumulation of methane in micropores without undergoing any phase transition over wide intervals of sub- and supercritical temperatures and pressures [62].

### 3.3. Calculation of Thermodynamic Functions of Adsorption Systems

#### 3.3.1. Differential Molar Isosteric Heat of Adsorption

The differential molar isosteric heat of adsorption, *q*_st_, is an important thermodynamic parameter, which describes the heat effects of adsorption processes [26,27,30,32,33,34]. By definition [30], the value of *q*_st_ is determined as the difference between the molar enthalpy of equilibrium gaseous phase *h*_g_ and differential molar enthalpy of adsorption system *H*_1_:

(4) $$q_{st}=h_{g}-H_{1}$$

As noted above, the study was intended to consider the effects associated with the nonideality of a gaseous phase and non-inertness of an adsorbent in terms of the approach by Bakaev [26]. Therefore, we calculated the differential molar isosteric heat of adsorption using an equation:

(5) $$q_{st}=-R\cdot Z\cdot {\left[\frac{\partial \left(lnP\right)}{\partial \left(1/T\right)}\right]}_{a}\cdot \left[1-{\left(\frac{\partial V_{a}}{\partial a}\right)}_{T}/\nu_{g}\right]-{\left(\frac{\partial P}{\partial a}\right)}_{T}\cdot \left[V_{a}-T\cdot {\left(\frac{\partial V_{a}}{\partial T}\right)}_{a}\right]$$

where *Z* = *P*⋅*ν*_g_/(*RT*) is the coefficient of compressibility of the equilibrium gas phase at pressure *P* (Pa) and temperature *T* (K); *ν*_g_ is the specific gas phase volume, m^3^/kg; *R* is the universal gas constant, J/(mol·K); *V*_a_ = *V*_0_/*m*_0_ is the reduced volume of the adsorbent-adsorbate system, cm^3^/g; and *V*_0_ and *m*_0_ are the volume and mass of the regenerated adsorbent, respectively. Thus, the Bakaev Equation (4) most fully takes into account the factors which affect the value of differential molar isosteric heat of adsorption: adsorption isothermal deformation (∂*V_a_*/∂*a*)*_T_*, temperature isosteric deformation (∂*V_a_*/∂*T*)*_a_*, the slopes of the isotherm of adsorption (∂*P*/∂*a*)*_T_* and isosteres [∂ln*P*/∂(1/*T*)]*_a_*, and the nonideality of a gas phase *Z* [26,27].

It was shown in [33] that in the conditions under consideration, the corrections for adsorption deformation of carbon adsorbents upon methane adsorption is minimal (~2–3%) and can be ignored in calculating *q*_st_. The data reported by Novikova [63] enabled us to evaluate the maximal value of isosteric temperature deformation (∂*V_a_*/∂*T*)_a_ and show that the term *T*⋅(∂*V_a_*/∂*T*)_a_ is much lower than *V_a_* in the studied temperature and pressure range. Therefore, we used Equation (5) without corrections for the adsorption-stimulated and thermal deformations of adsorbent:

(6) $$q_{st}=-R\cdot Z\cdot {\left[\frac{\partial \left(lnP\right)}{\partial \left(1/T\right)}\right]}_{a}-{\left(\frac{\partial P}{\partial a}\right)}_{T}\cdot V_{a}$$

**Figure 6:**
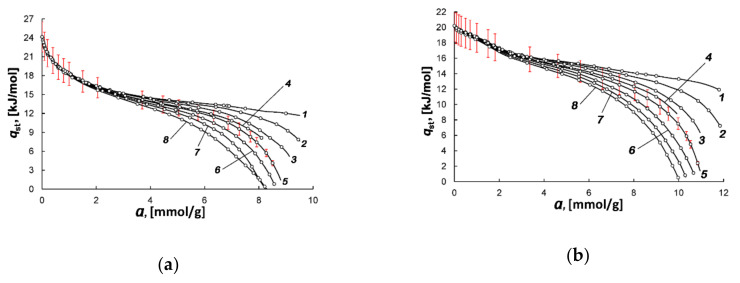
The differential molar isosteric heat of adsorption versus the value of methane adsorption in AC-4 (**a**) and AC-6 (**b**) at temperatures, K: 178 (*1*), 216 (*2*), 243 (*3*), 273.15 (*4*), 300 (*5*), 320 (*6*), 340 (*7*), 360 (8). Symbols show experimental data; solid curves are the results of approximation by Equation (6). The error bar is 10%.

Figure 6a,b show the functions *q*_st_ = *f*(*a*)_T_ for AC-4 and AC-6 calculated from the experimental isotherms of methane adsorption following to Equation (6) for different temperatures.

The dependences of *q*_st_(*a*) shown in Figure 6a,b are determined by the changes in the methane adsorption mechanisms in ACs as micropores become filled. Indeed, as can be seen from Figure 4a,b, at low pressures (up to 1 Pa), the carbon adsorbents tend to strongly adsorb methane molecules, which occupy a large portion of micropores by binding to high-energy adsorption sites. The high-energy micropores with a width close to the adsorbate molecule diameter with very high adsorbent fields and surface functional groups serve as such adsorption sites. For example, the snapshots and density profiles obtained upon the Grand Canonical Monte Carlo (GCMC) and Molecular Dynamics (MD) simulations of adsorption and dynamic behaviors of methane in the interior of constricted slit models of carbon adsorbents [64] provided evidence that at low pressures methane was adsorbed in the slits with a constriction width close to the molecular diameter, where one layer of adsorbed methane was formed. At low adsorbate loadings, the absolute value of *q*_st_ depends on the density of adsorption sites, which are the high-energy micropores and heteroatoms, including metal ions. The latter is associated with electrostatic interactions, which are likely to be of minor importance for methane, which has no dipole or quadrupole moments. In any case, the summary effect of these factors determines the lower value of *q*_st_ found for AC-6 (~20 kJ/mol) compared to AC-4 (~24 kJ/mol). AC-4 differed by a smaller percentage of narrow micropores (see Table 1) and lower content of oxygen than AC-6. Unlike AC-6, it contains metal ions (see Table 2). According to atomistic simulations [65], a steep initial decrease in *q*_st_ with *a* observed in AC-4 is conventionally attributed to heterogeneity in the adsorbent. More precisely, it occurs when the pore size distribution is skewed towards the large pore width, as in the case of AC-4. The reduced slope of *q*_st_ = *f*(*a*) observed at higher values of micropore loading for both adsorbents is likely a result of overlapping the impacts from the adsorbate−adsorbent and adsorbate−adsorbate interactions [65]. When the adsorbate−adsorbate attraction tends to dominate upon the process of methane accumulation, they lead to the formation of adsorption associates of methane molecules in micropores [62,66]. As a result, the heats of methane adsorption in AC-4 and AC-6 come closer together.

As follows from Figure 6a,b, the isosteric heat of adsorption is temperature-independent in the early stages of adsorption. However, with the increase in the value of adsorption, the curves *q*_st_ = *f*(*a*)_T_ fall as the high-energy adsorption sites become occupied, and the rate of fall of the curves depends on temperature. Therefore, one can observe a so-called “fan” of the curves, each of which correspond to a particular temperature (see Figure 6a,b). With a further increase in pressure, the curves describing the dependency of heats of adsorption diverge according to Equation (5) due to the variations in the temperature-dependent contributions from the compressibility of the gas phase Z and derivative (∂*P*/∂*a*)⋅*V_a_* related to the steepness of the adsorption isotherms in the *a*-*P* coordinates.

#### 3.3.2. Integral Heat of Methane Adsorption in the Activated Carbons

The methane adsorption/desorption processes are accompanied by the release/absorption of a large amount of heat, which degrades the storage capacity of ANG systems. These thermal effects are estimated by the values of integral heat of adsorption *Q*:

(7) $$Q\left(T\right)=\int_{0}^{a}q_{\mathrm{st}}{\left(a\right)}_{T=const}da$$

The integral heats of methane adsorption in AC-6 and AC-4 were calculated from the obtained data on isosteric heats of adsorption. The values of *Q* were used to evaluate the value of ∆*T*, which is a variation in temperature upon the heating-up/cooling of a 50 L model ANG system filled with AC-4 or AC-6 under adiabatic conditions. In the calculations, we used the specific heat capacity of activated carbons of 0.84 kJ/(kg·K) [67]. Table 3 summarizes the results of the calculations.

According to Table 3, the increase in temperature produced a decrease in the values of *Q*. The methane adsorption process in the ANG system based on AC-6, which is the more active adsorbent for methane, is accompanied by the release of a more significant amount of heat compared to that for AC-4. Additionally, the data in Table 3 testify that the heating-up of the ANG system is maximal under adiabatic conditions and low temperatures. At 178 K, ∆*T* achieved 69 and 59 K for the ANG systems based on AC-6 and AC-4, respectively. With increasing temperature, the heating-up reduces: at 360 K, ∆*T* is 39 and 33 K for the ANG systems based on AC-6 and AC-4, respectively.

#### 3.3.3. Differential Molar Isosteric Entropy of the Methane-AC Adsorption Systems

The entropy of adsorption system *S*_1_ is an essential thermodynamic state function, which gives insight into the state of adsorbate molecules in micropores, including their interactions in adsorption associates and with the adsorbent. The isosteric molar entropy of an adsorption system *S*_1_ can be calculated using a formula derived from Equation (4) relative to the equilibrium gas phase:

(8) $$S_{1}=s_{g}-\frac{q_{st}}{T}$$

**Figure 7:**
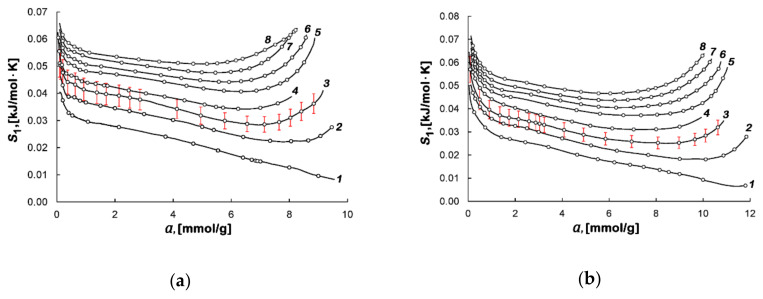
The differential molar entropy of the methane-AC-4 (**a**) and methane-AC-6 (**b**) adsorption systems versus the values of methane adsorption at temperatures, K: 178.00 (*1*), 216.00 (*2*), 243.00 (*3*), 273.15 (*4*), 300.00 (*5*), 320.00 (6), 340.00 (7), and 360.00 (*8*). Symbols indicate the experimental data; solid lines are the smoothing spline curves. The error bar is 10%.

Figure 7a,b show the calculated values of differential molar entropy of the adsorption systems based on AC-4 and AC-6.

Figure 7a,b demonstrate a steep initial decrease in the entropy at *a* < 0.5 mmol/g, which is attributed to the occupation of the most strongly adsorbing sites. In the region of intermediate values of adsorption, from 1 to 7 mmol/g, the decrease in the entropy slows down due to further loading of micropores and the formation of the adsorption associates caused by increasing mutual attraction between adsorbate molecules [66]. When micropore fillings are high, a sharp rise in curves *S*_1_(*a*) is observed, which can be interpreted as a result of the rearrangement of the adsorbate structure, leading to the formation of the denser methane associates. As was shown in [32,33], a further increase in methane adsorption leads to a weak local peak followed by a drop in the entropy at high fillings of micropores. These variations in the entropy are indicative of the completion of the formation of adsorption associates and subsequent rearrangement of adsorbed molecules caused by the increased repulsion between them.

#### 3.3.4. Differential Molar Isosteric Enthalpy of the Methane-AC Adsorption Systems

The enthalpy of an adsorption system, *H*_1_, is a thermodynamic state function, which determines an amount of energy, which the system can potentially transform into heat—in other words, the heat content:

(9) $$H_{1}=h_{g}-q_{st}$$

**Figure 8:**
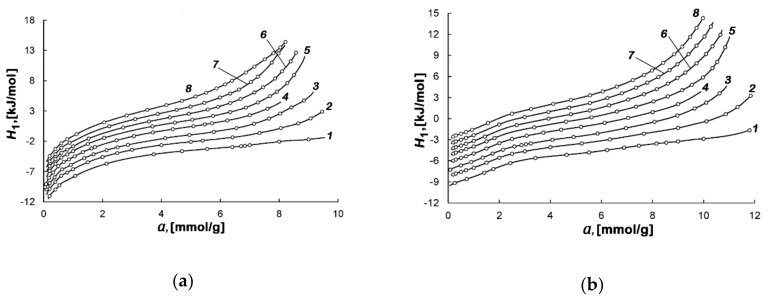
Dependence of the differential molar isosteric enthalpy of the methane-AC-4 (**a**) and methane-AC-6 (**b**) adsorption systems on the value of adsorption at temperatures, K: 178.00 (*1*), 216.00 (*2*), 243.00 (*3*), 273.15 (*4*), 300.00 (*5*), 320.00 (*6*), 340.00 (*7*), and 360.00 (*8*). Symbols indicate the experimental data; solid lines are the smoothing spline curves.

Figure 8a,b shows the results of the calculation of *H*_1_ for the studied adsorption systems at different temperatures.

As follows from Figure 8a,b, the enthalpy is dependent on temperature, and this dependence becomes stronger with the increase in the micropore fillings. The negative values of enthalpy *H*_1_ are determined by the reference level accepted for the standard state of the gas phase—*h*_g_.

**Figure 9:**
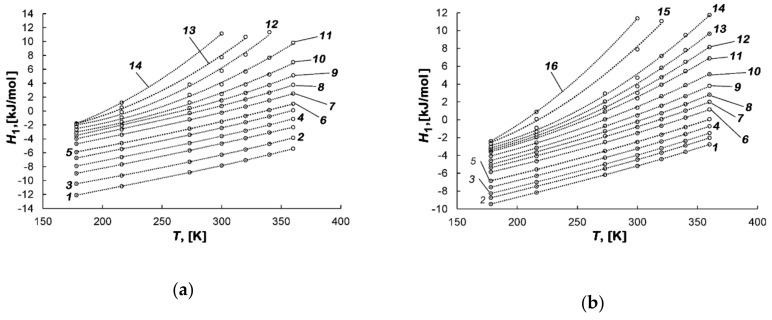
Temperature dependence of the differential molar enthalpy of the methane-AC-4 (**a**) and methane-AC-6 (**b**) adsorption systems at the values of adsorption *a*, mmol/g: 0.1 (*1*), 0.3 (*2*), 0.6 (*3*), 1 (*4*), 1.5 (*5*), 2 (*6*), 3 (*7*), 4 (*8*),5 (*9*), 6 (*10*), 7 (*11*), 7.8 (*12*), 8.3 (*13*), 8.8 (*14*) (**a**) and 0.1 (*1*), 0.6 (*2*), 1 (*3*), 1.5 (*4*), 2 (*5*), 3 (*6*), 4 (*7*), 5 (*8*), 6 (*9*), 7 (*10*), 8 (*11*), 8.5 (*12*), 9 (*13*), 9.5 (*14*), 10.5 (*15*), 11 (*16*) (**b**).

Figure 9a,b shows the temperature dependences of differential molar enthalpy of the methane-AC-4 and methane-AC-6 systems at various values of adsorption.

Figure 9a,b shows that, at low loading, the enthalpy of these systems increases almost linearly, which is caused by the temperature invariance *q*_st_ ≠ *f*(*T*) and the linear dependence of *h*_g_ on temperature. However, with the increase in the micropore fillings, the dependences *H*_1_(T) become nonlinear. The rate of their growth, *H*_1_’(*T*), increases with temperature, which is determined by the contribution from temperature-dependent compressibility of the gas phase Z and derivative (∂*P*/∂*a*)⋅*V_a_* in Equation (6) for *q*_st_ (see the fan of curves *q*_st_ = *f*(*a*) in Figure 6a,b).

The variations in *H*_1_ with *a* and *T* obtained from the experimental data for both methane-AC adsorption systems are consistent with those calculated for model micropores in many numerical experiments. As was found in [68] by using the continuous fractional component Monte Carlo algorithm, the growth of enthalpy at high micropore loadings close to saturation is caused by repulsive interactions arising between an introduced adsorbate molecule and both the previously adsorbed species and the adsorbent. The appearance of these intermolecular repulsive forces leads to the rearrangement of the adsorption associates.

#### 3.3.5. Differential Molar Isosteric Heat Capacity of the Methane-AC Adsorption Systems

In contrast to the specific heat capacity of the bulk phase, the specific heat capacity of any adsorption system is dependent not only on pressure, temperature but on the value of adsorption. Therefore, a comprehensive thermodynamic description of an adsorption system includes the differential molar isosteric heat of adsorption—*C_a_*. The values of *C_a_* were calculated using the Kirchhoff equation derived by the differentiation of Equation (4) over the temperature:

(10) $$C_{a}={\left(\frac{\partial H_{1}}{\partial T}\right)}_{a}={\left(\frac{\partial h_{g}}{\partial T}\right)}_{a}-{\left(\frac{\partial q_{\mathrm{st}}}{\partial T}\right)}_{a}$$

**Figure 10:**
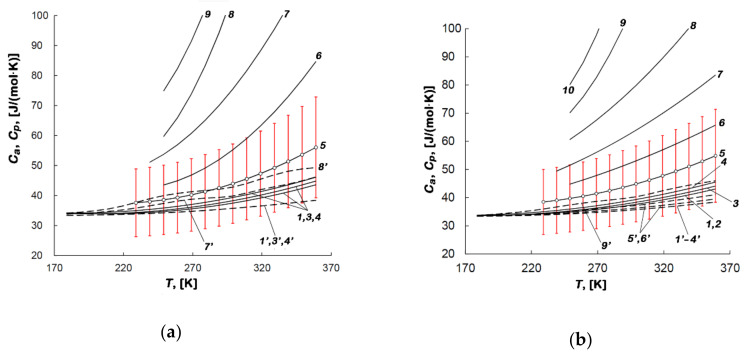
Temperature dependences of the differential molar isosteric heat capacities of the methane-AC-4 (**a**) and methane-AC-6 (**b**) adsorption systems and the gas phase (numbering with stroke) at the values of methane adsorption, mmol/g: 0.1 (*1*), 0.3 (*2*), 1.0 (*3*), 2.0 (*4*), 4.0 (*5*), 6.0 (*6*), 7.0 (*7*), 7.8 (*8*), 8.3 (*9*). The error bar is 30%.

Figure 10a,b shows the temperature dependencies of the differential molar isosteric heat capacities of the methane-AC-4 and methane-AC-6 adsorption systems, respectively.

Figure 10a,b shows that the heat capacity of the adsorption systems is identical in magnitude to the isobaric heat capacity of the gas system (see 1, 2, 3, 4 and 1’, 2’, 4’ curves). However, when the temperatures are above 220 K, the differences become more significant, and the heat capacity of the adsorption system exceeds the values of *C*_p_. Furthermore, with the increase in the methane adsorption values and temperature, the heat capacity also increases. Moreover, the *C_a_*(*T*) curves diverge more strongly as the temperature increases.

As follows from Figure 10a,b, the isosteric heat capacities of the methane-AC-4 and methane -AC-6 adsorption systems differ significantly from each other at the same adsorption values. Indeed, at *a* ~ 8 mmol/g and 270 K, the isosteric heat capacity of the methane-AC-4 system is 45% more than that in the adsorption system with AC-6. Therefore, one can conclude that the isosteric heat capacity is lower when methane is adsorbed in AC-6 with a higher volume of micropores and less content of carbon species compared to AC-4.

According to [32], which summarized the thermodynamic functions of a large number of gas/microporous material adsorption systems, in most cases, the *C_a_*(*T*) dependence exhibited a local maximum at elevated temperatures. When adsorption achieved the high values, this maximum shifted towards lower temperatures, as was the cases of Xe [69] and CH_4_ adsorption [70,71] in NaX zeolites, and CH_4_ adsorption in the microporous silicon carbide-derived activated carbon labeled as AUK [33]. Therefore, we suggest that these regularities are general and will be observed in the studied adsorption systems with the increase in the values of methane adsorption, pressure, and temperature. Such features of the *Ca*(*T*) dependences cannot be caused by the gaseous phase, since these dependencies significantly diverge; for example, curves 7 and 7’. The adsorption-induced deformation of activated carbon, which is the component of the adsorption system, is also not responsible for this effect, since the changes in its crystal structure are imperceptible. The only logical explanation for this effect is a change in the state of adsorbed molecules, namely the formation of adsorption associates in micropores, which is confirmed by the results of molecular dynamics simulations [34,62,66].

## 4. Conclusions

The activated carbons were synthesized from peat of high degree of metamorphism in three stages: carbonization, thermochemical K_2_S activation at 1053–1133 K, and steam activation at 1173 K. The stage of steam activation performed for two different periods resulted in two different AC samples: AC-4 and AC-6. The data of SEM, X-ray, and adsorption investigations revealed that the most activated carbon possessed the largest micropore volume and specific BET surface. The increase in the activation time caused the changes in the chemical composition and crystallinity. A combination of these changes determines a higher adsorption capacity of AC-6 with respect to methane. The results indicate that a detailed comparative analysis of the adsorption performance, characteristics of porous structure (SEC), and data on phase and chemical composition for activated carbons prepared from various precursors under different activation conditions is needed to establish a precise correlation between textural and adsorption behaviors. This correlation is essential for determining a criterium for an optimal adsorbent for ANG storage.

The general thermodynamic equation by Bakaev made it possible to calculate the differential molar isosteric heats of methane adsorption in AC-4 and AC-6 at pressures up to 25 MPa in the temperature range from 178 to 360 K. The differential molar isosteric entropy, enthalpy, and heat capacity evaluated as the functions of temperature and methane adsorption for both methane-AC adsorption systems are affected by heterogeneous porous, phase and chemical structure of the adsorbents. It should be noted that despite the differences in absolute values, the variations in the thermodynamic functions with temperature and adsorption values reflect the general pattern of changes in the state of adsorbed molecules upon the adsorption process in the heterogeneous microporous carbons: from binding with the high-energy adsorption sites to the formation of the adsorption molecular associates, and their subsequent rearrangement close to saturation.

The heating-up of both the ANG systems calculated from the integrated heat of adsorption was found to be maximal under adiabatic conditions and low temperatures. The magnitudes of the temperature changes of the ANG system based on AC-4 upon the adsorption (charging) process are lower than those found for that with AC-6. When the temperature of the adsorption process grows, the heating-up of both adsorption systems decreases.

The isosteric heat capacity of the methane adsorption system based on AC-4 with a lower degree of activation and higher energy of adsorption exceeded that for the system with AC-6. When the value of adsorption is close to 8 mmol/g, and the temperature is 270 K, the difference between the values of *C_a_* for the systems based on AC-4 and AC-6 amounts to ~ 45%.

Finally, the evaluation of the key thermodynamic quantities is essential for selecting an efficient adsorbent, designing both the ANG systems and heat-exchange facilities to achieve the maximum of both storage density and deliverable capacity.

## Figures and Tables

**Table 1:** The parameters of the porous structure of the peat-derived activated carbons.

Sample	*E*_0_, kJ/mol	*W_0_*, cm^3^/g	*x*_0_, nm	*S*_BET_, m^2^/g	*W*_t_, cm^3^/g	*S*_meso_, m^2^/g	*W*_meso_, cm^3^/g
AC-4	20.6	0.48	0.58	957	0.72	105	0.24
AC-6	19.1	0.60	0.63	1334	0.70	96	0.10

**Table 2:** Elemental chemical composition of the peat-derived activated carbons.

Sample	Elements (wt.%)										
	C	O	K	S	Si	Al	Cl	Ca	Fe	Mg	B
AC-4	74.9	10.7	0.8	3.0	2.4	0.6	0.6	1.4	0.3	0.18	4.9
AC-6	60.8	23.7	0.5	1.7	10.2	1.2	0.6	1.3	-	-	-

**Table 3:** Thermodynamic characteristics of the adsorbed natural gas (ANG) systems based on AC-4 and AC-6.

AC	*T*, K	178.00	216.00	243.00	273.15	300.00	320.00	340.00	360.00
**AC-4**	*P*, MPa	1.0	3.4	6.2	5.1	13.0	16.0	18.0	23.0
	*a*, mmol/g	9.6	9.6	9.2	8.1	9.0	8.7	8.3	8.3
	*Q,* kJ/kg	141.4	135.8	126.6	115.9	116.9	110.4	104.1	99.3
	*∆T*, K	58.8	51.9	45.2	46.0	39.4	37.0	35.2	32.6
**AC-6**	P, MPa	1.0	4.0	5.7	5.5	15.0	19.0	21.0	23.0
	*a*, mmol/g	11.9	11.9	11.0	10.0	11.1	10.9	10.4	10.1
	*Q,* kJ/kg	182.1	174.1	159.4	147.7	149.3	140.9	133.8	128.1
	∆*T*, K	69.3	58.6	53.8	53.6	44.4	41.4	40.0	38.7
